# Spider assemblages associated with different crop stages of irrigated rice agroecosystems from eastern Uruguay

**DOI:** 10.3897/BDJ.6.e24974

**Published:** 2018-05-03

**Authors:** Leticia Bao, Juaquín Ginella, Mónica Cadenazzi, Enrique A. Castiglioni, Sebastián Martínez, Luis Casales, María P. Caraballo, Álvaro Laborda, Miguel Simo

**Affiliations:** 1 Unidad de Entomología. Facultad de Agronomía. Universidad de la República. Garzón 780. CP 12900, Montevideo, Uruguay; 2 Sección Entomología. Facultad de Ciencias. Universidad de la República. Iguá 4225. CP 11400, Montevideo, Uruguay; 3 Departamento de Biometría Estadística y Computación. Estación Experimental Mario Cassinoni, EEMAC. Facultad de Agronomía. Universidad de la República, Ruta 3 km 363, Paysandú, Uruguay; 4 Centro Universitario Regional del Este, CURE. Universidad de la República. Ruta 9 y Ruta 15, Rocha, Uruguay; 5 Laboratorio de Patología Vegetal, Instituto Nacional de Investigación Agropecuaria, Ruta 8 Km 281, CP33000, Treinta y Tres, Uruguay

**Keywords:** Agroecology, Araneae, diversity, guilds composition, rice crop

## Abstract

The rice crop and associated ecosystems constitute a rich mosaic of habitats that preserve a rich biological diversity. Spiders are an abundant and successful group of natural predators that are considered efficient in the biocontrol of the major insect pests in agroecosystems. Spider diversity in different stages of the rice crop growth from eastern Uruguay was analysed. Field study was developed on six rice farms with rotation system with pasture, installed during intercropping stage as cover crop. Six rice crops distributed in three locations were sampled with pitfall and entomological vaccum suction machine. Sixteen families, representing six guilds, were collected. Lycosidae, Linyphiidae, Anyphaenidae and Tetragnathidae were the most abundant families (26%, 25%, 20% and 12%, respectively) and comprised more than 80% of total abundance. Other hunters (29%), sheet web weavers (25%) and ground hunters (24%) were the most abundant guilds. Species composition along different crop stages was significantly different according to the ANOSIM test. The results showed higher spider abundance and diversity along the crop and intercrop stages. This study represents the first contribution to the knowledge of spider diversity associated with rice agroecosystem in the country.

## Introduction

The study of biological diversity associated with agroecosystems has focused the attention of biologists in the last decades to produce sustainable crops. One of them has been the cultivation of rice, which is the most ancient form of intensive agriculture in the world (Fernando 1977). During 2016, more than 159 million hectares were cultivated in more than 100 countries (FAOSTAT 2016). Irrigated rice crops are seasonal temporary wetland ecosystems with a variable degree of agronomical management (Bambaradeniya et al. 2004). Throughout a single cultivation period, rice agroecosystem presents three major temporary ecological phases: aquatic, semiaquatic and dry (Fernando 1995).

Uruguay produced 1,348,301 tonnes of rice, becoming the seventh exporter country inthe world during the 2013/2014 season (ACA 2015). According to the Khush classification (Khush 1984), Uruguay cultivates rice in irrigated environments with a standing layer of water of 5 to 10 cm of depth. Rice cultivation in the country is mostly based on a rotational production system with perennial pastures, consisting in two years of rice crop, followed by four years of pastures, integrated with livestock production (ACA 2013). One of the more important cultivated areas is the eastern region of the country, located within the Bañados del Este, a wetland region which belongs to an internationally protected area by the RAMSAR convention. In this region of the country, rice cultivation area is commonly surrounded by others crops or pasture areas or remnants of native ecosystems like patches of riparian forests and wetlands (PROBIDES 1999).

The rice crop and associated ecosystems (natural environments or other crops) constitute a rich mosaic of habitats that preserve a high biological diversity (Roger 1996). This disturbance promotes intensive changes in the ecotones like processes of colonisation, migration, reproduction and higher rates of growth of organisms (Bambaradeniya 2000). Furthermore, the flood production system provides a temporal environment that is beneficial for the conservation of invertebrates and vertebrate species (Heong et al. 1991, Schoenly et al. 1996). Currently, the International Rice Research Institute (IRRI) aims to develop ecological engineering methods to strengthen the diversity of natural enemies and to increase the ecosystem services they provide (Norton et al. 2010). Some important components of this diversity are certain arthropod groups which participate as regulators of insect pest populations (Roger 1996), giving an ecological service provided by biodiversity of the rice ecosystem itself (Heinrichs and Barrion 2004, Naeem et al. 1999). Spiders constitute a megadiverse order with high value as biological control agents against the major insect pests in agroecosystems (Riechert and Lockley 1984, Norma-Rashid et al. 2014, Pompozzi et al. 2014). The ecosystem service provided by spiders as generalist predators in agroecosystems is supported in part by the availability of alternative habitats from which they take refuge and recolonise the crop after cultivation and the following growth stages (Hibbert and Buddle 2008).

A few studies related to spider diversity in agroecosystems and adjacent environments have been carried out in Uruguay (Simó et al. 2011, Jorge et al. 2013). Despite the fact that rice has been grown in Uruguay for so many years, there are no data about the spider fauna associated with this crop in the country and the impact on insect pest communities. Considering the role of this group, the knowledge of the assemblages of spider species present in the different rice phenological stages is crucial for the ecological management of the crop.

In the present paper, we study the spider diversity in different stages of the rice crop from eastern Uruguay, with the aim of identifying the changes along the crop cycle and to provide baseline information as a tool for evaluation of the impact of management practices on crop sustainability.

## Materials and methods

**Study area and crop management**

**Figure 1.**
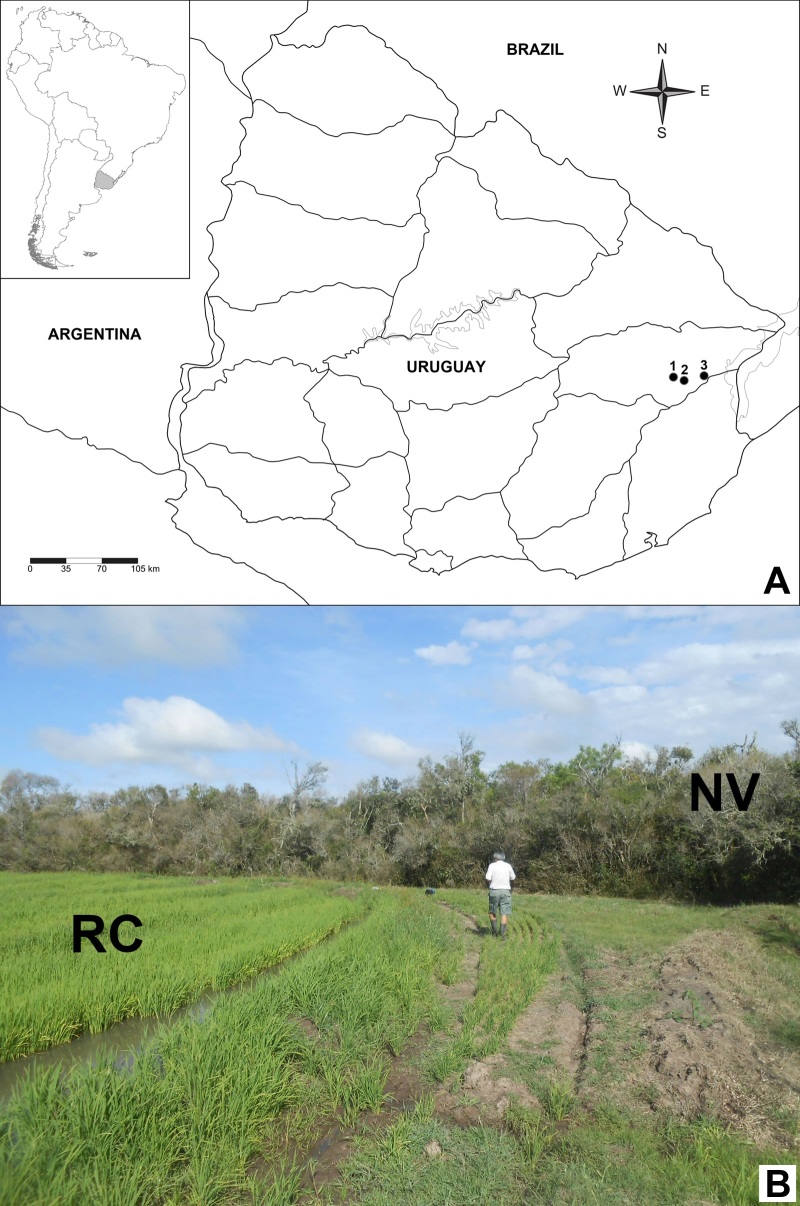
A) Sampling locations Julio María Sanz (1), El Tigre (2) and General Enrique Martínez (3). B) View of the rice crop (RC) usually surrounded by native vegetation patches (NV)

The main area for rice production in Uruguay is located in the eastern region of the country (ACA 2015). It belongs to Laguna Merín basin, with rice and livestock farming as main production activities. The field study was conducted in rice farms of first and second year crop in a system rotation with pasture, installed during intercropping stage as cover crop. Sampling periods were performed considering intercropping and the phenological stages of the crop where the main rice pests are detected: post-seeding, tillering and grain filling. Post-seeding is an early stage during the first 20 to 30 days of the crop where the early seedling growth occurs. Tillering is a flooded stage that comprises the vegetative growth of the plant. Grain filling is also a flooded stage where immature grains of rice arise. Pastures were composed of a mix of *Lotus corniculatus* L. (Fabaceae), *Lolium multiflorum* Lam. (Poaceae) *and Trifolium repens* L. (Fabaceae). Native vegetation patches present in the study area correspond to the type riparian forest habitat with an average vegetation height of 4 metres. The most common floristic composition of this area is the hydrophitic species as *Phyllantus sellowianus* Müll. Arg. (Phyllanthaceae) immediate to the water line and an intermediate edge of transition species to pasture represented by *Eryththrina cristagalli* L. (Fabaceae), *Scutia buxifolia* Reiss. (Rhamnaceae), *Celtis tala* Gillies ex Planch. (Cannabaceae), *Schinus longifolius* (Lindl.) Speg. (Anacardiaceae), *Myrcianthes cisplatensis* (Cambess.) O. Berg (Myrtaceae), *Tillandsia* sp. (Bromeliaceae) (Muñoz et al. 2011). Three collecting sites were selected: Julio María Sanz (33°11'54.99"S, 54°5'12.30"W), El Tigre (33°13'27.80"S, 53°59'38.84"W) and General Enrique Martínez (33°12'8.15"S, 53°50'47.98"W) located in Treinta y Tres Department, eastern Uruguay (Fig. 1). According to the Köppen-Geiger classification, Uruguay belongs to the Cfa climate type, which corresponds to temperate climate without a dry season and the hottest month (January) with the temperature above 22°C (Peel et al. 2007). The soil type is a melanised solod of the “La Charqueada” Soil Unit. The crop was treated only with herbicide previous to sowing; no insecticide was applied on rice and pasture (Altamirano et al. 1976).

**Spider collection and data analysis**

From November 2013 to November 2015, two rice paddies on each of the three collecting sites were sampled seasonally (one rice paddy of first and other of second year), resulting in six rice paddies in the whole work. Spiders were sampled with pitfall traps and an entomological vacuum suction machine. Fifteen pitfall traps were installed for each crop and set for a week. Each trap consisted of a 400 ml cup containing 100 ml conservative mix (8.5 volumes of distilled water, 1.5 volumes of acetic acid 4%, 1 volume NaCl). Ground and vegetation from the surrounded area of each pitfall trap (3 to 4 metres away) was sampled during one minute with the vacuum suction machine (fifteen samples per paddy). The collected material was kept in 70% ethanol. Specimens were identified at family and species/morphospecies level using keys and taxonomic revisions from literature (Grismado et al. 2014). Vouchers were deposited in the arachnological collection of Facultad de Ciencias, Universidad de la República, Uruguay. Only adults were considered for species/morphospecies identification. Guilds were assigned following Cardoso et al. (2011). EstimateS 9.1.0 (Collwell 2013) was used to calculate species accumulation curves for each collecting method and analytical richness estimators (Chao 1, Jacknife 1 and Bootstrap) in order to evaluate the sampling effort. Total number of spiders per sampling moment were compared using generalised linear-mixed model with Poisson distribution (PROC GLIMMIX, S.A.S. Institute 2009). Means were separated using Tukey-Kramer (p<0.05). To test statistic differences intaxonomic composition between the sampling moments, we used ANOSIM and SIMPER analysis performed with PAST 3.14 software (Hammer et al. 2001).

## Results

A total of 16 families, 61 species/morphospecies and six guilds of spiders were registered (Table 1). From the 2088 spiders collected, 945 were adults (45%) and 1143 juveniles (55%). The most abundant spider families were Lycosidae, Linyphiidae, Anyphaenidae and Tetragnathidae (26%, 25%, 20% and 12%, respectively) that represented more than 80% of total relative abundance (Table 2).

*Hisukattus transversalis* Galiano, 1987, *Lycosa auroguttata* (Keyserling, 1891), *Lobizon corondaensis* (Mello-Leitão, 1941), *Agalenocosa velox* (Keyserling, 1891), *Sphecozone ignigena* (Keyserling, 1886), *Camillina chilensis* (Simon, 1902), *Mazax ramirezi* (Rubio & Danişman, 2014) and *Arachosia magna* are registered for the first time for Uruguay (Table 1).

**Figure 2.**
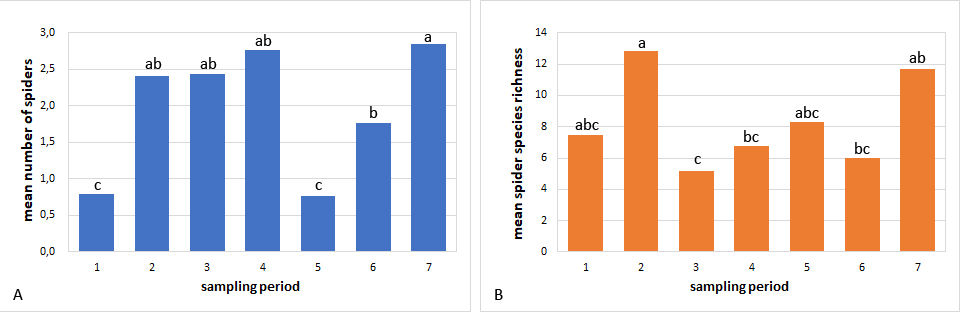
A) Mean number of spiders per sample. Different letters indicate significant differences compared by Tukey-Kramer test (F=24.22, df=562, p<0.0001). B) Mean spider species richness. Different letters indicate significant differences compared by Tukey_Kramer test (F=7.16, df=6, p<0.0036). Sampling periods: 1:post-seeding,; 2 and 5: tillering, 3 and 6: grain filling; 4 and 7: intercrop.

Spider abundance adjusted to Poisson distribution and presented statistical differences between sampling periods, showing higher values in the intercrop stages and the lower values immediately after seeding (F= 24.22, df=562 p>0.0001, Fig. 2A). The discrete variable (number of spiders per sample) was adjusted to Binomial, Negative Binomial and Poisson distribution. The indicators used to compare the adjustments were the Akaike (AIC), Bayesian (BIC) criteria and the logarithm of the Maximum Likelihood (-2LMV). Poisson distribution had the best values for all the indicatiors mentioned above in all cases. Species richness per sampling period was higher at tillering of the first year and intercrop of the second year sampling (F=7.16, df=6, p<0.0036; Fig. 2B). Considering sampling done during crop presence, tillering stage richness values were higher than the grain filling stage.

**Figure 3.**
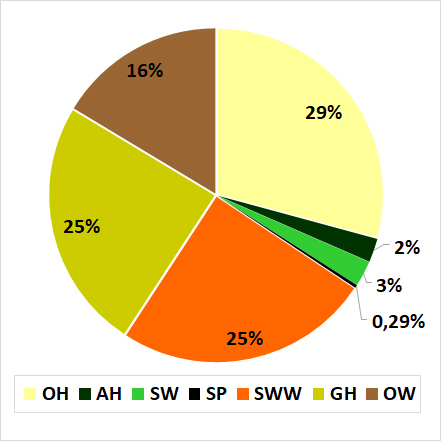
Spider guilds relative abundances for the whole collecting period. OH: other hunters, AH: ambush hunters, SWW: sheet web weavers, SP: specialists, SW: space weavers, GH: ground hunters, OW: orb web weavers.

Other hunters, sheet web weavers and ground hunters were the more abundant guilds with 29%, 25% and 24% of relative abundance respectively (Fig. 3).

**Figure 4.**
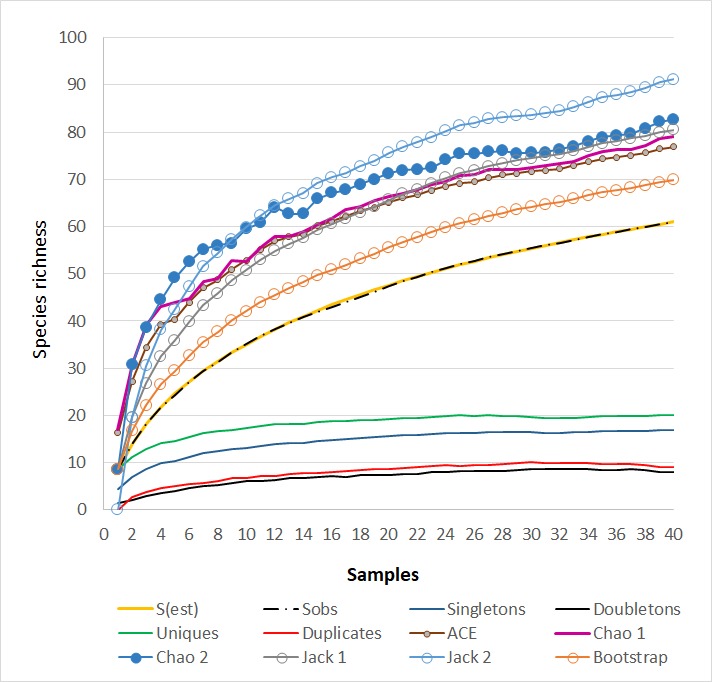
Species accumulation curves of observed (S) and corrected richness (S est: 500 randomisations), *singletons*, *doubletons*, *uniques* and *duplicates* from the forty samples from different sampling moments: 1-6: post-seeding, 7-12, 23-28: tillering, 13-18, 29-34: grain filling, 19-22, 35-40: intercrop.

Species accumulation curve was non-asymptotic, indicating that there could be additional species to be sampled (Fig. 4). Richness estimators showed that at least 67% of the total expected species were sampled (Incidence based estimators: Jack 2: 91.17, 67%; Chao 2: 82.67, 74%; Bootstrap 69.85, 87%; Jack 1: 80.5, 76%; Abundance based estimator Chao 1: 79.04; 77%). Singletons represented 27.8% of the species collected, doubletons 13.1%, uniques 32.8% and duplicates 14.7%.

**Figure 5.**
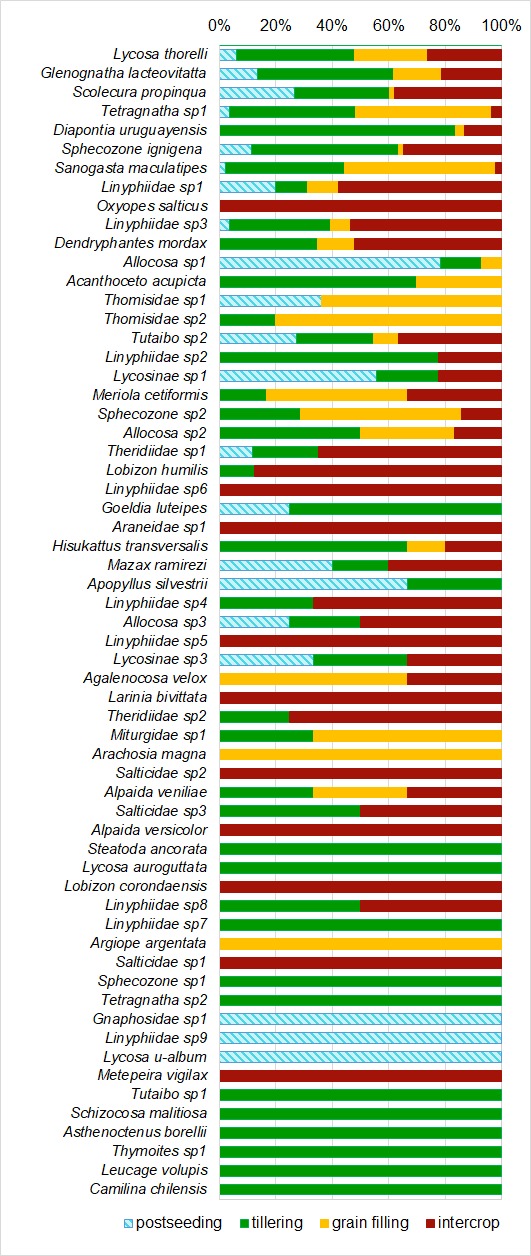
Relative abundances of the more abundant species according to crop stage.

According to the SIMPER test comparing between collecting moments, *Lycosa thorelli* (Keyserling, 1877), *Glenognatha lacteovitatta* (Mello-Leitao, 1944), *Scolecura propinqua* (Millidge, 1991), *Tetragnatha* sp. 1, *Diapontia uruguayensis* (Keyserling, 1877), *Sphecozone ignigena* (Keyserling, 1886) and *Sanogasta maculatipes* (Keyserling, 1891) contributed to 57% of the observed dissimilarity (Suppl. material 1). Relative abundances of species by crop stage showed different patterns according to the species considered (Fig. 5). *Arachosia magna* (Rubio and Ramírez, 2015), *Acanthoceto acupicta* (Nicolet 1849) and *Apopyllus silvestri* (Simon 1905) were collected only in the rice crop. Meanwhile *Oxyopes salticus* (Hentz 1845), *Alpaida versicolor* (Keyserling 1877), Salticidae sp. 1 and Salticidae sp. 2, were collected only during the intercrop stage. Species composition for collecting moments were significantly different according to the ANOSIM test (R=0.544, p=0.0001 Jaccard index, R=0.433, p=0.0001 Morisita index).

## Discussion

Total number of spiders per sample was lower in the early crop stages and increasing to the end of the crop cycle. Recently tilled fields had low vegetation complexity and represent a critical period for predator’s establishment (Ryptstra et al. 1999). The recovery of spider populations after disturbances in the field is achieved by reproduction, but immigration of surrounding habitats is also very important (Thorbek and Topping 2005). Therefore, surrounding habitats like pastures, other crops and riparian vegetation patches could serve as a reservoir of species that can recolonise the rice crop after the tillage or other management disturbances (Thorbek and Bilde 2004). Considering species richness during the crop cycle, the higher values observed at the tillering stages could be explained by the high intensity of spider colonisation from neighbouring environments. Beltramo et al. 2006 observed in sosybean crops from Argentina, that after soil disturbance, spiders with aerial dispersion promote recolonisation from surrounding habitats.

In this study, we confirm that rice crops serve as reservoirs for spider species that where recorded at different regional environments.

*A. magna* was reported in grasses and near streams from Argentina (Rubio and Ramírez 2015). According to this, the species was registered only for the grain filling stage, when rice plants are in flooded ground.

*A. velox* was registered in flooded grasslands from Argentina (Piacentini 2014). Similarly, in the rice crop, the species was collected in grain filling (flooded area) and intercrop stages (pastures).

*L. corondaensis* was reported for woodlands neighbouring grasslands from Argentina (Piacentini and Grismado 2009). Althougth a few specimens were collected in this study, they were registered in pasture and flooded stages of the rice crop.

*Mazax ramirezi* was collected in pitfall traps for grasslands from Buenos Aires, Argentina. In this study, all the exemplars were also obtained with this type of traps and the species was found throughout the rice cycle.

*Lobizon humilis* (Mello-Leitao, 1944) (Lycosidae) was registered mainly during the intercrop stage. The species was reported from Argentina (Piacentini and Grismado 2009) and prefers open grasslands (Rubio et al. 2008). Other records from data collection in Uruguay are associated with rocky hills and wetlands (Simó et al. 2015). *Sphecozone ignigena* (Linyphiidae) has been collected in rice crops in southern Brazil (Rodrigues et al. 2008). In this study, it was registered in all crop and intercrop stages, being more abundant during rice presence.

*Sanogasta maculatipes* (Anyphaenidae) was more abundant at tillering and grain filling stages in the rice cultivation, as it was also reported from Brazilian crops (Rodrigues et al. 2008). The species constructs refuges on foliage and grasses (Ramírez 2003) which explains its scarcity in the post-seeding stage of the crop, where vegetation complexity is scarce. *Diapontia uruguayensis* (Lycosidae) was registered throughout the whole cycle, but it was more abundant during the tillering stage when water has just arrived to the crop. This agrees with the fact that the species usually lives in association with water streams or flooded soils (Piacentini et al. 2017). The presence of *Asthenoctenus borellii* Simon, 1897 (Ctenidae) and *Apopyllus silvestri* (Gnaphosidae) during postseed and tillering stages was expected, considering these species have been reported in Uruguay from native but also from disturbed environments (Simó et al. 2000, Costa et al. 2006)

*Glenognatha lacteovitatta* (Tetragnathidae) was reported for alfalfa and wheat crops from Argentina (Armendano and González 2010, Armendano and González 2011). The species was found in all the stages surveyed in this study which suggests it colonises the initial stages of the crop from the surrounding habitats. *Goeldia luteipes* (Titanoecidae) was similar, being recorded in post-seeding and tillering stages.

The family Linyphiidae presented high species diversity and it was the second more abundant in this study. This result agrees with the results of Rodrigues et al. (2013) in rice crops from southern Brazil. *Sphecozone ignigena* was reported from rice crops in southern Brazil (Rodrigues et al. 2013) and, in the present study, it was recorded along the crop and the intercrop stages. This suggests that this species and other linyphiids represent an important part of the spiders colonising rice seedlings after crop installation.

The range percentage obtained for the richness estimators was nearly 70% to 87%, indicating that a comprehensive inventory would be reached (Cardoso 2009) and additional species are pending sampling.

Except for sensing web spiders, all the guilds proposed by Cardoso et al. (2011) were registered in this study. Other hunters (29%), sheet web weavers (25%) and ground hunters (25%) were the most abundant guilds in the crop. Other hunters were mostly represented by Anyphaenidae and Salticidae. Previous studies related to spider guilds structure in rice crop (Uetz et al. 1999, Rodrigues et al. 2008) registered ground hunters and sheet web weavers as the more abundant guilds. In the present study, they ranked as second and third most abundant guilds, mostly composed of Lycosidae and Linyphiidae, respectively. These families are commonly abundant in agroecosystems in many parts of the world and are mentioned as potential pest control agents (Nyffeler and Sunderland 2003). According to Jocqué and Dippenaar-Schoeman (2007), lycosids are supposed to have co-evolved with grasslands, which could explain the abundance of this group in the rotation system with pastures used in Uruguay, representing a fourth part of the total abundance at family level (Table 2). Additionally, the use of pitfall traps in this study can also explain the greater abundance of this family. These traps are considered a good method for collecting dwelling spiders (Green 1999). Linyphiids also represented a fourth part of the total abundance at family level in this study (Table 2). They are abundant in moderate temperatures and high humidity regions, where they spin sheet webs in tall herbs or close to the ground (Nyffeler and Sunderland 2003). The fourth mostabundant guild was the group of orb web weavers mostly represented by *Glenognatha lacteovitatta* (Tetragnathidae). This species was reported as a common species in alfalfa and wheat in Argentina (Armendano and González 2010, Armendano and González 2011). Some species of *Glenognatha* construct webs close to the soil surface (Hormiga and Döbel 1990) and specimens of this genus were reported in rice crops in Arkansas (Heiss and Meisch 1985). Ballooning present in linyphiid and tetragnathid species represent a good ability for dispersion, colonisation and survival to water contact (Hayashi et al. 2015), which is an advantageous characteristic for living in rice paddy fields.

This study represents the first contribution to the knowledge of spider diversity associated with rice crop agroecosystems in Uruguay. The results showed a high spider abundance and diversity throughout the crop and intercrop stages. Future research should focus on successional changes in the mosaic of landscapes of the region and evaluate the effects of management strategies on biodiversity, in order to promote its conservation and assure a sustainable rice crop production through natural biological control.

## Supplementary Material

Supplementary material 1 Dissimilarity Matrix SIMPER Test Data type: Excel file
Brief description: Dissimilarity Matrix of SIMPER Test obtained with PAST 3.14 software (Hammer et al. 2001).
File: oo_198147.xlsx L.Bao, M.Simó

## Figures and Tables

**Table 1.** Families and species collected in rice fields.

**Family**	**Species/morphospecies**
Anyphaenidae	*Acanthoceto acupicta*
	*Arachosia magna*
	*Sanogasta maculatipes*
Araneidae	*Alpaida veniliae*
	*Alpaida versicolor*
	*Argiope argentata*
	Araneidae sp1
	*Larinia bivittata*
Corinnidae	*Mazax cf ramirezi*
Ctenidae	*Asthenoctenus borellii*
Gnaphosidae	*Camilina chilensis*
	*Apopyllus silvestri*
	*Gnaphosidae* sp1
Linyphiidae	*Linyphiidae* sp1 to sp9
	*Scolecura propinqua*
	*Sphecozone ignigena*
	*Sphecozone* sp1
	*Sphecozone* sp2
	*Tutaibo* sp1
	*Tutaibo* sp2
Lycosidae	*Agalenocosa velox*
	*Allocosa* sp1
	*Allocosa* sp2
	*Allocosa sp3*
	*Diapontia uruguayensis*
	*Lobizon corondaensis*
	*Lobizon humilis*
	*Lycosa cf thorelli*
	*Lycosa u-album*
	*Lycosinae* sp1
	*Lycosinae* sp2
	*Lycosa auroguttata*
	*Schizocosa malitiosa*
Miturgidae	Miturgidae sp1
Oxyopidae	*Oxyopes salticus*
Pholcidae	Pholcidae sp1
Salticidae	*Hisukattus transversalis*
	*Dendryphantes mordax*
	Salticidae sp1
	Salticidae sp2
	Salticidae sp3
Tetragnathidae	*Glenognatha lacteovitatta*
	*Leucage volupis*
	*Tetragnatha* sp1
	*Tetragnatha* sp2
Theridiidae	*Steatoda ancorata*
	Theridiidae sp1
	Theridiidae sp2
	*Thymoites* sp1
Thomisidae	Thomisidae sp1
	Thomisidae sp2
Titanoecidae	*Goeldia luteipes*
Trachelidae	*Meriola cetiformis*

**Table 2.** Family relative abundances during different crop stages.

Family	Sampling moment								
	post-seeding	tillering 1	grain filling 1	intercrop 1	tillering 2	grain filling 2	intercrop 2	Subtotal	%
Anyphaenidae	25	116	144	6	24	67	33	415	19.88
Araneidae	0	6	12	30	7	12	9	76	3.64
Corinnidae	2	0	0	0	1	0	2	5	0.24
Ctenidae	1	0	0	0	0	0	0	1	0.05
Gnaphosidae	3	1	0	0	1	0	0	5	0.24
Linyphiidae	100	129	26	91	37	11	122	516	24.71
Lycosidae	30	197	53	32	65	59	114	550	26.34
Miturgidae	0	1	2	0	0	0	0	3	0.14
Oxyopidae	0	4	1	1	1	1	24	32	1.53
Pholcidae	0	0	1	0	0	0	0	1	0.05
Salticidae	9	35	49	2	4	11	25	135	6.47
Tetragnathidae	23	106	24	18	25	39	10	245	11.73
Theridiidae	2	8	16	9	4	1	11	51	2.44
Thomisidae	2	3	1	4	0	26	7	43	2.06
Titanoecidae	1	0	0	0	3	0	0	4	0.19
Trachelidae	0	0	1	0	1	2	2	6	0.29
	198	606	330	193	173	229	359	2088	100

## Hosting institution

Unidad de Entomología. Facultad de Agronomía. Universidad de la República. Garzón 780. CP 12900. Montevideo. Uruguay.
